# Molecular weight control in organochromium olefin polymerization catalysis by hemilabile ligand–metal interactions

**DOI:** 10.3762/bjoc.12.131

**Published:** 2016-07-04

**Authors:** Stefan Mark, Hubert Wadepohl, Markus Enders

**Affiliations:** 1 Anorganisch-Chemisches Institut, Heidelberg University, Im Neuenheimer Feld 270, D-69120 Heidelberg, Germany

**Keywords:** chromium single-site catalysts, olefin polymerization, ultra-high molecular weight polyethylene

## Abstract

A series of Cr(III) complexes based on quinoline-cyclopentadienyl ligands with additional hemilabile side arms were prepared and used as single-site catalyst precursors for ethylene polymerization. The additional donor functions interact with the metal centers only after activation with the co-catalyst. Evidence for this comes from DFT-calculations and from the differing behavior of the complexes in ethylene polymerization. All complexes investigated show very high catalytic activity and the additional side arm minimizes chain-transfer reactions, leading to increase of molecular weights of the resulting polymers.

## Introduction

Chelate ligands with both, a strongly coordinating moiety and a weakly coordinating donor function allow the stabilization of vacant coordination sites at metal centers and may act as placeholders for external substrates. The concept of hemilabile ligands has been introduced in 1979 [1] and has been applied for the development of improved transition metal catalysts [2–7]. The donor–acceptor interaction of the hemilabile moiety with the metal center should be weak enough to allow the displacement by a substrate, which should itself be transformed during the catalytic reaction. The bonding ability of internal or external stabilizing ligands in relation to the substrate plays a crucial role. In contrast to external donors, a special feature of a hemilabile donor is the fact that only one stabilizing ligand per metal center is available. Consequently, a large excess of external donors (e.g., solvent molecules, substrates, additives, etc.) may displace a relatively strong intramolecular donor function. Several examples for olefin polymerization catalysts with hemilabile ligands are known and the impact of the hemilabile group on the polymerization behavior can be immense [8–12]. Examples are the switching from polymerization to trimerization selectivity [13] or the suppression of chain termination by weak interactions with fluorine substituents [14–17]. The interaction with fluorine atoms from fluorinated borate anions have also shown to play a role in olefin polymerization [18–24].

Cyclopentadienyl (Cp)-based chromium complexes exhibit very good ethylene polymerization properties, when the coordination sphere of the chromium center is completed by an additional ligand. Improved stability and hence polymer productivities are obtained when the donor is tethered to the Cp ring [25–36]. However, the tethered donor usually does not act as a hemilabile ligand as it remains coordinated during the catalytic process. Many cyclopentadienyl (Cp) ligands where an additional neutral donor function is covalently bonded have been reported [37–39]. Some of those donors bind strongly, others weakly, to a particular transition metal ion. Examples, which are related to the work described here, are Cp ligands with olefinic [40–52] or with a nitrile side arm [53–54].

**Scheme 1:**
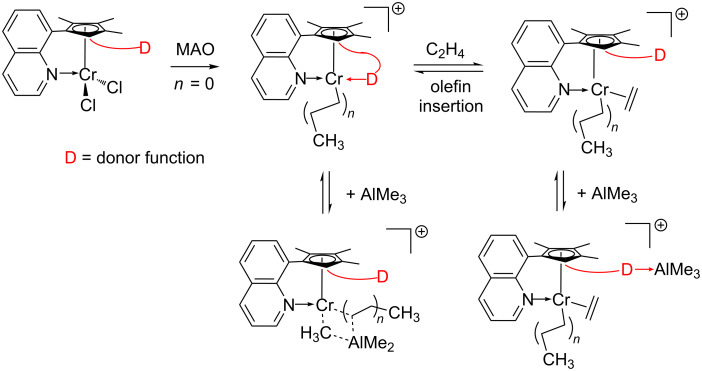
Influencing catalyst stability, olefin coordination and chain-transfer reactions by hemilabile donor functions.

We have recently described how external modifiers combined with Cp-chromium polymerization catalysts influence the chain-termination process and hence the molecular weight of the produced polyethylene [55]. This paper describes our results with covalently linked modifiers and their influence on the ethylene polymerization behavior. There is neither experimental nor theoretical evidence for a substantial beta-H elimination or beta-H transfer in such catalyst systems so that chain termination is dominated by chain-transfer reactions to the aluminum based co-catalysts [56–57]. Consequently, any component, which modulates the interaction of Al–alkyls with the catalyst center, can influence the molecular weight of the resulting polyethylene. The concept of the present investigation is sketched in Scheme 1. The pre-catalysts feature covalently bonded neutral donor functions (D) which do not interact with the coordinatively saturated chromium centers. The coordination ability of the chosen donors D range from very weak (organofluorine) to medium (olefinic or aryl, respectively) and strong (nitrile). Activation with methylaluminoxane (MAO) leads to the formation of monomethyl complexes and the active species is a cationic alkylchromium complex with one remaining „vacant“ coordination site. This site can bind one of the following donors: ethylene, internal labile donor D, alkylaluminium compound, etc. If the internal donor binds, the cationic chromium center is stabilized but ethylene can displace the donor and insert into the chromium alkyl bond leading to polyethylene. In the presence of aluminum alkyls like trimethylaluminum chain termination may occur by addition of AlMe_3_ to the cationic chromium alkyl species. A simultaneous coordination of the internal donor D and alkylaluminum is not possible or at least very unlikely so that interaction of the hemilabile donor D can suppress chain transfer. Another possibility is that the donor D directly interacts with AlMe_3_, which also reduces the chain-transfer rate. Both types of interaction lead to an increase in molecular weight of the polyethylene.

**Scheme 2:**
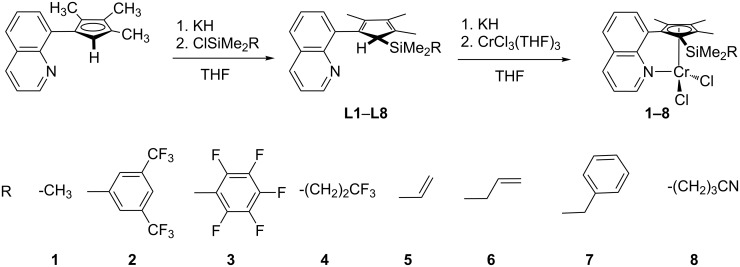
Synthesis of ligands **L1**–**L8** and chromium complexes **1**–**8**.

We have synthesized a number of ligands as presented in Scheme 2 and evaluated the interaction of the donor units with the Lewis acidic chromium center by DFT methods. The synthesized complexes where then tested in ethylene polymerization in order to evaluate the influence of the hemilabile donor in terms of catalyst activity and molecular weight of the polymer.

## Results and Discussion

It is well known that early transition metal single-site polymerization catalysts can interact with organofluorine groups [15,17,20,22]. In FI-type catalysts the interaction of fluorine substituents with the catalyst center leads to a suppression of chain termination so that living olefin polymerization is possible [15]. The experimental verification of such an interaction was demonstrated by NMR of an MAO activated complex [17]. Consequently, we envisaged the synthesis of ligands where an organofluorine substituent is connected by a side arm with suitable length. In addition to that we choose side arms with olefinic groups, a benzyl unit and a stronger nitrile donor group, respectively. The synthesis of the new ligand derivatives and the corresponding Cr complexes follows known procedures (Scheme 2) [29,58]. The key step for the introduction of the hemilabile donor function is the electrophilic attack of a chlorosilane derivative at quinolyl-functionalized cyclopentadienides (Cp^Q^). We used the trimethyl Cp^Q^ derivative as this leads to a single acidic proton in the ligands **L1**–**L8**. By this procedure we could introduce side arms with fluorine (**L2**–**L4**) or olefinic donor groups (**L5**, **L6**) as well as benzyl (**L7**) or nitrile (**L8**) moieties. Deprotonation with potassium hydride and subsequent reaction with chromium trichloride leads to the chromium complexes as green-blue solids in yields ranging from 21% (**3**) to 81% (**6**).

**Figure 1:**
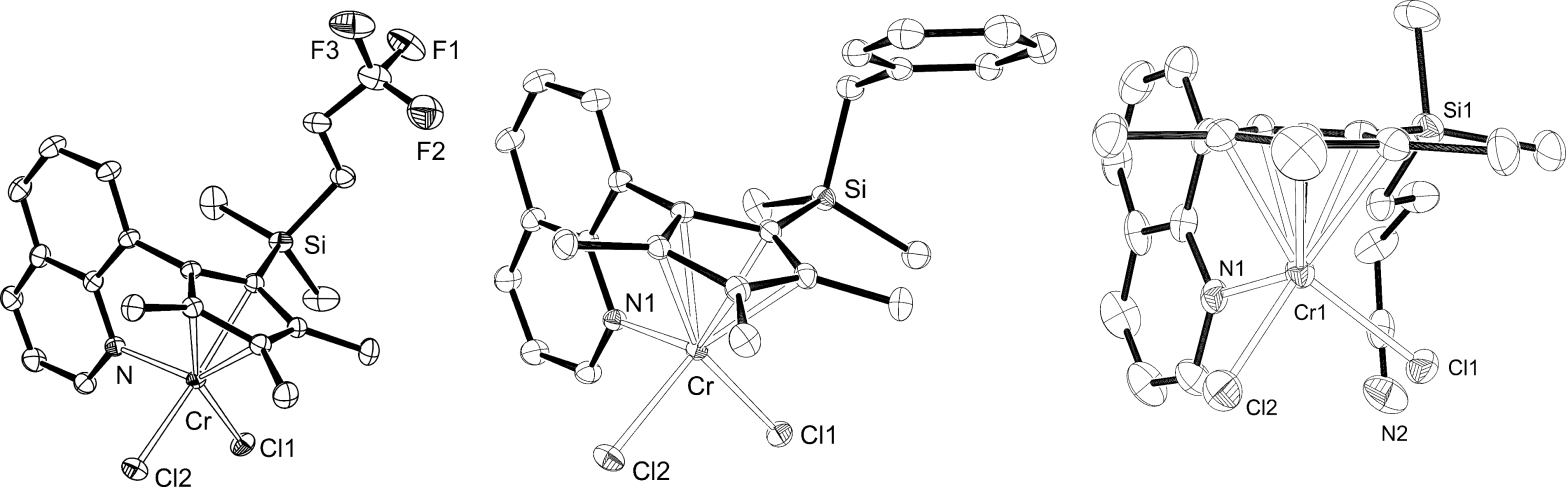
Solid-state molecular structures of selected complexes **4**, **7** and **8** (left, middle and right, respectively). Probability level: 50%. Hydrogen atoms and carbon labels are omitted for clarity.

The ligand **L3** as well as the pre-catalysts **4–8** were studied by single crystal X-ray analysis. A selection of molecular structures is presented in Figure 1 and details of the structure determination are presented in Table S1 (see Supporting Information File 1). Due to the rigid and predefined geometry of the Cp^Q^ ligand, the coordination environment around the chromium centers is very similar in all cases and in line with previously published structures of such complexes [29,59–60].

As the metal center in the pre-catalysts **1**–**8** is coordinatively saturated, the additional side arm functionality cannot interact with the metal. However, after activation with a co-catalyst, the complexes become cationic with a vacant coordination site so that the side arms could interact with the metal centers. Crystals of an activated complex could not be obtained. Only very few examples are known where X-ray diffraction data could be obtained from the active form of chromium polymerization catalysts [61–63]. However, DFT calculation is well suitable for studying such interactions. By such methods it is not only possible to estimate the binding energy but also to compare it with that of ethylene or interaction with solvent molecules like toluene.

**Figure 2:**
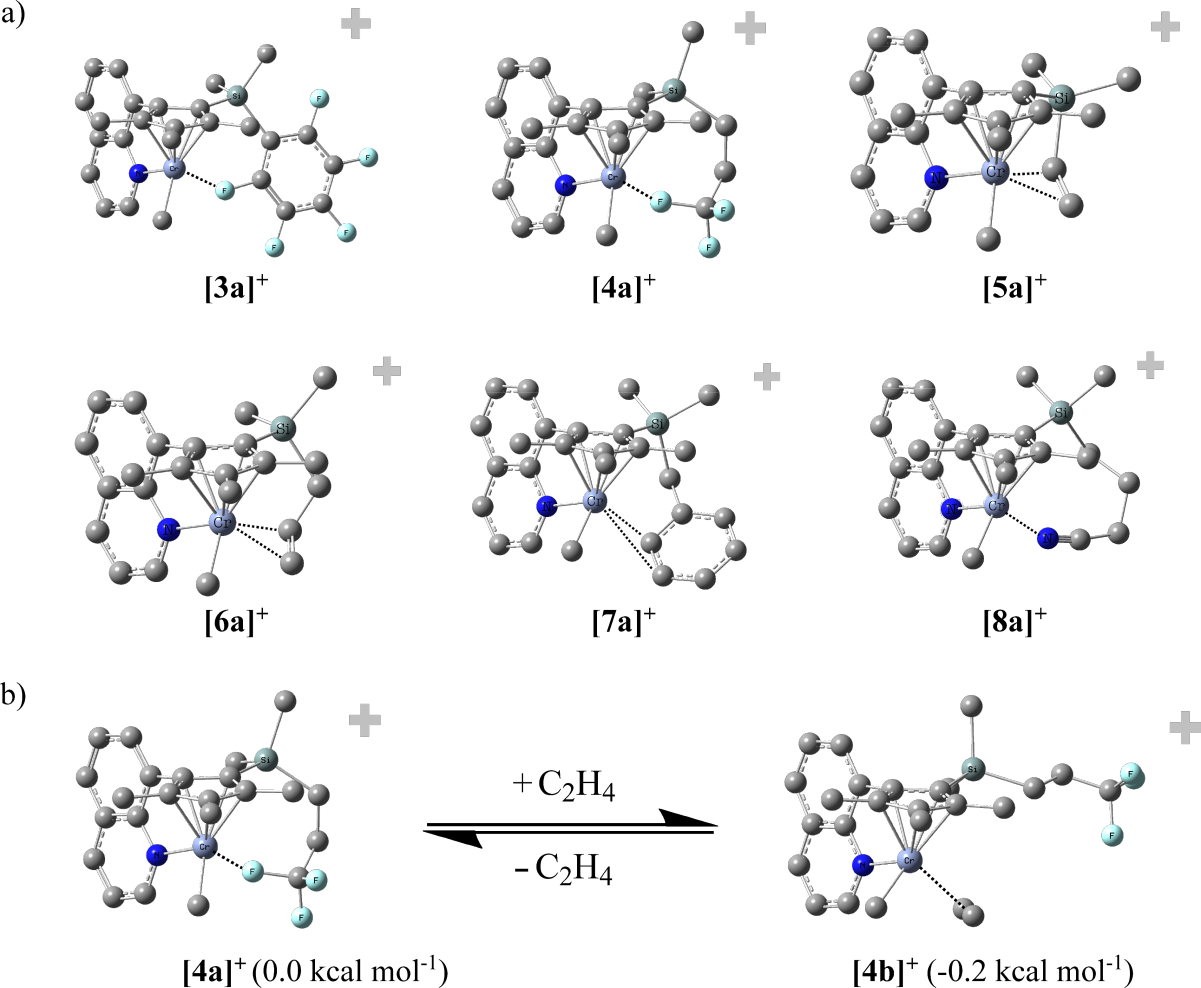
a) Calculated structures of cationic chromium complexes. Chromium–donor distances [Å]: **3a^+^** [2.21], **4a^+^** [2.24], **5a^+^** [2.53/2.51], **6a^+^** [2.75/2.46], **7a^+^** [2.58/3.17], **8a^+^** [2.06]. b) Displacement of hemilabile donor by ethylene under formation of the ethylene complex **4b^+^**. (Energies for the formation of the analogous ethylene complexes **3b^+^** [−1.3], **5b^+^** [−4.5], **6b^+^** [+2.9], **7b^+^** [−2.9], **8b^+^** [+15.2].)

All DFT calculations were performed with the B3LYP functional and the 6-311g* basis set. This theoretical level has shown to reproduce well paramagnetic NMR shifts in such compounds [58,64–65]. As we compare only relative energies of the complexes, the errors in the absolute energy values will compensate considerably. As a model for the activated catalysts we calculated the cationic monomethyl complexes **1a^+^**–**8a^+^**. The complexes with ligands with a suitable geometry for intramolecular coordination indeed showed minima structures (as shown by the absence of imaginary frequencies) where the functionalized side-arms interact with the metal center (complexes **3a^+^–8a^+^**, see Figure 2, for the analogous complexes **1a^+^** and **2a^+^**, respectively, no reasonable minima structures were obtained). We did not consider the conformers where the growing chain is on the “other” site (i.e., oriented in the direction of the Si substituent). From earlier theoretical work on related chromium complexes we know that the chain can move easily from one site to the other so that the hemilabile donor may interact easily [57].

The Cr–F distances in **3a^+^** and **4a^+^** are with 2.24 Å and 2.21 Å, respectively, in the range of non-covalent Cr–F interactions as exemplified by a cationic chromium complex with BF_4_^−^ anion (2.37 Å) [66] and a typical covalent Cr–F bond (~1.95 Å) [67].

According to calculations the distances of the two carbon atoms of the coordinating unit to the Cr centers in **5a^+^**, **6a^+^** and **7a^+^** are approximately 2.5 Å (shortest of the two Cr–C distances). No X-ray data are available for olefin complexes of chromium in oxidation state +3 whereas solid state molecular structures of Cr^0^ and Cr^1+^ complexes have been reported with C–Cr distances of 2.1 Å–2.2 Å (Cr^1+^) [68–69] and 2.3 Å–2.4 Å (Cr^0^) [70–71], respectively.

As an alternative to the intramolecular coordination of the hemilabile donor to the vacant coordination site at the chromium center, a dimerization of the cationic methylchromium fragment could occur and examples of such dicationic dimers have been reported [72–75]. We were able to identify local minimum structures of dimers of compounds **3a^+^**–**8a^+^** but the calculated energies lie considerably higher compared to monomeric forms. Consequently, the saturation by the weak hemilabile donor is energetically preferred over dimerization. Another possible interaction is the coordination of Al–alkyls to the chromium centers. This has been addressed in detail in our previous work, where we could show that the energies of such adducts are similar compared to chromium complexes with olefin coordination [55,57]. For complex 8a^+^, however, the situation is different: the interaction energy of the nitrile group with the chromium center leads to an energy gain which is 15.2 kcal mol^−1^ higher compared to the energy of the corresponding ethylene complex. Consequently, the ethylene can hardly displace the nitrile. However, addition of Al–alkyls leads to a strong interaction of the Al center with the nitrile group so that ethylene can coordinate. Related to this behavior is a report of an acetonitrile-stabilized chromium complex which upon activation with MAO leads to a highly active catalyst and even the addition of up to 4 equivalents of acetonitrile to the catalyst solution did not lead to lower catalyst activities [76].

### Polymerization results

The ethylene polymerization behavior of all new chromium complexes has been evaluated and compared with the performance of the known derivative **1** as well as with zirconocene dichloride (Cp_2_ZrCl_2_). The results are summarized in Table 1 and the exact procedure is described in the experimental part (Supporting Information File 1). The pre-catalysts were activated with PMAO, which is a non-hydrolytically prepared variant of MAO from the company Akzo Nobel (also called PMAO-IP for “polymeric MAO-improved properties”) [77]. All polymerizations were conducted at atmospheric ethylene pressure. The catalytic activities are very high for all derivatives ranging from 1400 to 3900 g polyethylene per mmol catalyst per hour. The lowest activities are obtained with the derivatives with fluorine substituents (complexes **2**–**4**, entries 2–4 in Table 1) whereas all other complexes show considerably higher activities in the range from 2800–3900 g (PE) mmol^−1^ (cat) h^−1^. Polymerization under only 1 bar of ethylene pressure may lead to artifacts coming from limitations of ethylene transport into the solution and this may lead to biased turnover numbers. More interesting in terms of the concept of this work is the molecular weight of the polymers. The derivative **1** leads to a molecular weight of 530 000 g mol^−1^. Introduction of the 3,5-bis(trifluoromethyl)phenyl group lowers the molecular weight drastically to 90 000 g mol^−1^. In this derivative the electron-withdrawing CF_3_ groups cannot coordinate to the cationic Cr center in the active catalyst form. Apparently the electron withdrawing C_6_H_3_(CF_3_)_2_ substituent leads to lower molecular weight. However, when the fluoro substituents are able to coordinate (complexes **3** and **4**, respectively) the polymer molecular weight is much higher compared to the results obtained with **2** and slightly higher compared to **1**. When the side-arm functionalities possess better donor properties the molecular weight increases considerably up to the UHMW-PE range (entries 5–8, Table 1). With pre-catalysts **5** or **6** it is also possible that the side arm (vinyl or allyl side arm, respectively) is incorporated into the polymer but we cannot verify this by our experimental data.

**Table 1:** Results of the ethylene-polymerization tests with complexes **1–8** and Cp_2_ZrCl_2_ as catalyst precursors.

entry^a^	catalyst	N_cat_ [μmol]	activity [g·mmol^−1^·h^−1^]	*M*_w_^b^ [10^3^ g·mol^−1^]	PE [g]	polym.- time [min]	[*M*_w_/*M*_n_]	degr. of cryst.^c^ [%]	*T*_m_^c^ [°C]

1	**1**	4.42	3240	530	2.87	12	3.1	65	135.5
2	**2**	6.37	1590	90	3.37	20	3.2	–^d^	132.5
3	**3**	6.88	1450	660	2.32	14	2.2	62	133.0
4	**4**	7.82	1910	610	4.48	18	3.5	66	135.5
5	**5**	4.30	3130	900	3.37	15	3.5	59	135.5
6	**6**	7.68	2780	1 070	4.99	14	4.0	56	132.5
7	**7**	3.76	3940	1 140	2.96	12	3.0	54	133.5
8	**8**	4.14	3560	1 450	2.46	10	4.9	64	133.0
9	Cp_2_ZrCl_2_	10.30	2330	600	4.78	12	2.4	57	132.5

^a^Standard conditions: co-catalyst: PMAO (7% solution in toluene), Al:Cr = 1000:1, room temperature, 150 mL of toluene, atmospheric pressure, all reactions were performed with identical flasks and stirring bars. ^b^GPC-measurements. ^c^DSC measurements, for details see Supporting Information File 1. ^d^The determined crystallinity was unexpectedly high which could be due to artifacts. Therefore, this value is not tabulated.

**Figure 3:**
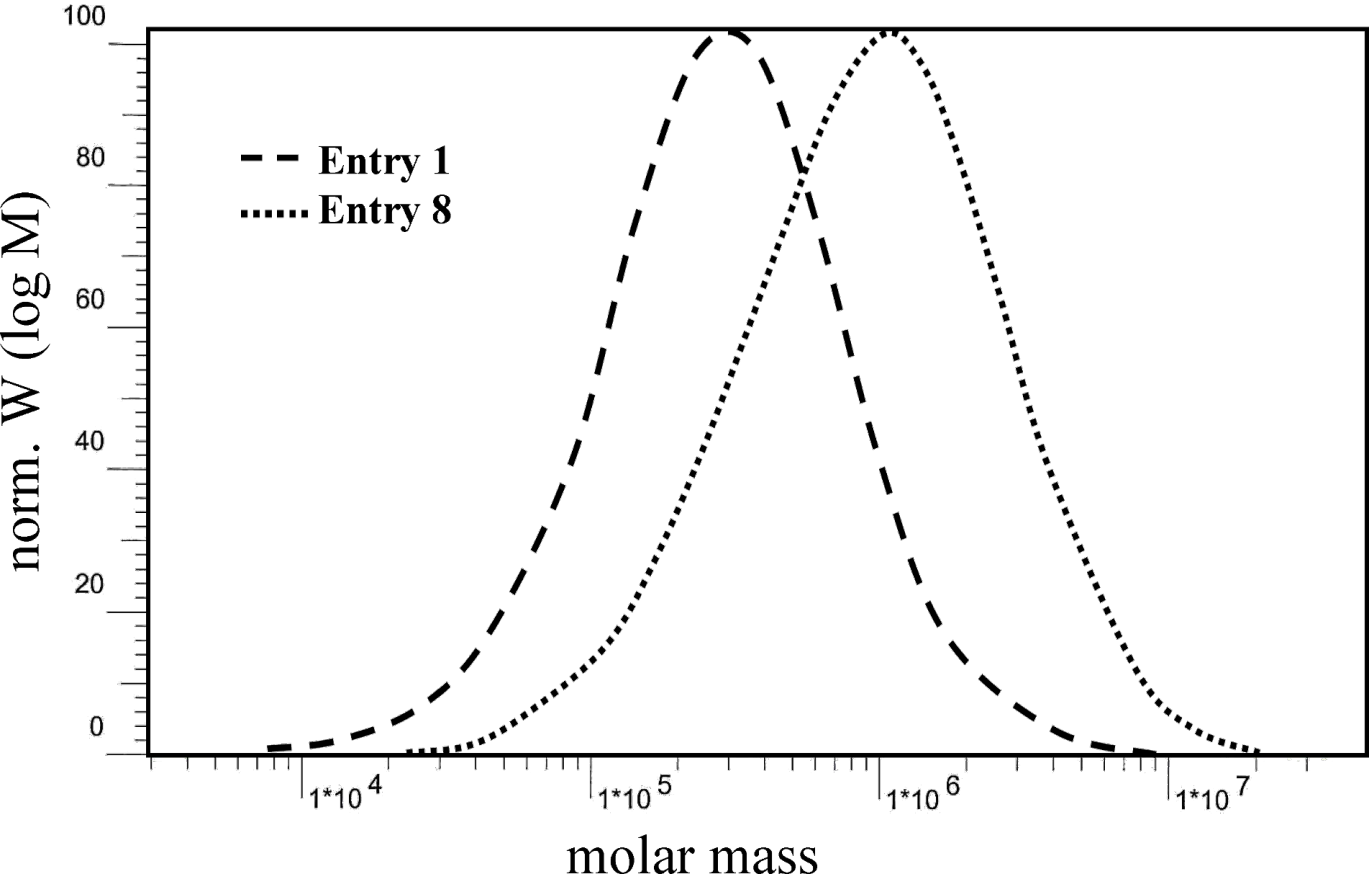
Comparison of GPC traces of polyethylene produced by **1**/PMAO and **8**/PMAO respectively (entries 1 and 8 in Table 1).

Figure 3 shows the effect on molecular weight of the produced polyethylene when comparing the known pre-catalyst derivative **1** with the new derivative **8**. As mentioned in the Introduction, the dominating chain termination pathway in olefin polymerization with Cp-chromium catalysts is chain transfer to aluminum alkyls and our results clearly show that this process can be suppressed efficiently by using the donor functions in the side arms of the silyl substituents. It has already been shown that external modifiers are also able to suppress chain termination. However, much higher amounts of such modifiers are necessary in order to give considerable effects [55,78].

## Conclusion

We have shown that weak donor groups, which are covalently bound to the metal complex can efficiently modulate chain termination processes in chromium-catalyzed ethylene polymerization catalysis. These hemilabile donors are able to protect coordinatively unsaturated metal centers against coordination of aluminum alkyls and hence reduce chain transfer to aluminum. On the other hand, the donors are weak enough in order to be displaced by ethylene monomers so that the insertion polymerization can proceed with high turnover numbers. With this concept, it is possible to tune the catalyst behavior in terms of the molecular weight they produce without lowering their very high catalytic activity.

## Supporting Information

File 1 Experimental part.

## References

[1] Jeffrey J C, Rauchfuss T B (1979). Inorg Chem.

[2] Slone C S, Weinberger D A, Mirkin C A (1999). The Transition Metal Coordination Chemistry of Hemilabile Ligands. Progress in Inorganic Chemistry.

[3] Lindner E, Speidel R, Fawzi R, Hiller W (1990). Chem Ber.

[4] Bader A, Lindner E (1991). Coord Chem Rev.

[5] Okuda J (1994). Comments Inorg Chem.

[6] Braunstein P, Naud F (2001). Angew Chem, Int Ed.

[7] Weng Z, Teo S, Hor T S A (2007). Acc Chem Res.

[8] Wang C, Ma Z, Sun X-L, Gao Y, Guo Y-H, Tang Y, Shi L-P (2006). Organometallics.

[9] Tshuva E Y, Groysman S, Goldberg I, Kol M, Goldschmidt Z (2002). Organometallics.

[10] Qian Y, Huang J, Bala M D, Lian B, Zhang H, Zhang H (2003). Chem Rev.

[11] Flores J C, Chien J C W, Rausch M D (1994). Organometallics.

[12] Müller C, Lilge D, Kristen M O, Jutzi P (2000). Angew Chem, Int Ed.

[13] Deckers P J W, Hessen B, Teuben J H (2001). Angew Chem, Int Ed.

[14] Chan M C W, Kui S C F, Cole J M, McIntyre G J, Matsui S, Zhu N, Tam K-H (2006). Chem – Eur J.

[15] Mitani M, Mohri J-i, Yoshida Y, Saito J, Ishii S, Tsuru K, Matsui S, Furuyama R, Nakano T, Tanaka H (2002). J Am Chem Soc.

[16] Chan M C W (2008). Chem – Asian J.

[17] Bryliakov K P, Talsi E P, Möller H M, Baier M C, Mecking S (2010). Organometallics.

[18] Ruwwe J, Erker G, Fröhlich R (1996). Angew Chem, Int Ed Engl.

[19] Sun Y, Spence R E v H, Piers W E, Parvez M, Yap G P A (1997). J Am Chem Soc.

[20] Karl J, Erker G, Fröhlich R (1997). J Am Chem Soc.

[21] Horton A D, Orpen A G (1991). Organometallics.

[22] Siedle A R, Newmark R A, Lamanna W M, Huffman J C (1993). Organometallics.

[23] Yang X, Stern C L, Marks T J (1994). J Am Chem Soc.

[24] Chen E Y-X, Marks T J (2000). Chem Rev.

[25] Thomas B J, Noh S K, Schulte G K, Sendlinger S C, Theopold K H (1991). J Am Chem Soc.

[26] Liang Y, Yap G P A, Rheingold A L, Theopold K H (1996). Organometallics.

[27] White P A, Calabrese J, Theopold K H (1996). Organometallics.

[28] Döhring A, Göhre J, Jolly P W, Kryger B, Rust J, Verhovnik G P J (2000). Organometallics.

[29] Enders M, Fernández P, Ludwig G, Pritzkow H (2001). Organometallics.

[30] Ikeda H, Monoi T, Ogata K, Yasuda H (2001). Macromol Chem Phys.

[31] Mani G, Gabbaï F P (2004). Angew Chem.

[32] Zhang H, Ma J, Qian Y, Huang J (2004). Organometallics.

[33] Randoll S, Jones P G, Tamm M (2008). Organometallics.

[34] Zhang L, Gao W, Tao X, Wu Q, Mu Y, Ye L (2011). Organometallics.

[35] Sieb D, Baker R W, Wadepohl H, Enders M (2012). Organometallics.

[36] Romano D, Ronca S, Rastogi S (2015). Macromol Rapid Commun.

[37] Siemeling U (2000). Chem Rev.

[38] Butenschön H (2000). Chem Rev.

[39] Müller C, Vos D, Jutzi P (2000). J Organomet Chem.

[40] Buzinkai J F, Schrock R R (1987). Organometallics.

[41] Okuda J, Zimmermann K H (1988). J Organomet Chem.

[42] Kohl F X, Dickbreder R, Jutzi P, Müller G, Huber B (1989). Chem Ber.

[43] Miguel-Garcia J A, Maitlis P M (1990). J Chem Soc, Chem Commun.

[44] Lehmkuhl H, Näser J, Mehler G, Keil T, Danowski F, Benn R, Mynott R, Schroth G, Gabor B, Krüger C (1991). Chem Ber.

[45] Ogasa M, Mallin D T, Macomber D W, Rausch M D, Rogers R D, Rollins A N (1991). J Organomet Chem.

[46] Erker G, Aul R (1991). Chem Ber.

[47] Zimmermann K H, Pilato R S, Horvath I T, Okuda J (1992). Organometallics.

[48] Okuda J, Zimmermann K H, Herdtweck E (1991). Angew Chem, Int Ed Engl.

[49] Okuda J, Zimmermann K H (1992). Chem Ber.

[50] Alt H G, Jung S H, Thewalt U (1993). J Organomet Chem.

[51] Spence R E v H, Piers W E (1995). Organometallics.

[52] Galakhov M V (1998). Chem Commun.

[53] Leong W L J, Garland M V, Goh L Y, Leong W K (2009). Inorg Chim Acta.

[54] Pinkas J, Gyepes R, Kubišta J, Horáček M, Lamač M (2011). J Organomet Chem.

[55] Mark S, Kurek A, Mülhaupt R, Xu R, Klatt G, Köppel H, Enders M (2010). Angew Chem, Int Ed.

[56] Döhring A, Jensen V R, Jolly P W, Thiel W, Weber J C (2001). Organometallics.

[57] Xu R, Klatt G, Enders M, Köppel H (2012). J Phys Chem A.

[58] Fernández P, Pritzkow H, Carbó J J, Hofmann P, Enders M (2007). Organometallics.

[59] Enders M, Fernández P, Mihan S, Pritzkow H (2003). J Organomet Chem.

[60] Enders M, Kohl G, Pritzkow H (2004). Organometallics.

[61] Bazan G C, Rogers J S, Fang C C (2001). Organometallics.

[62] MacAdams L A, Buffone G P, Incarvito C D, Rheingold A L, Theopold K H (2005). J Am Chem Soc.

[63] Theopold K H (1998). Eur J Inorg Chem.

[64] Liimatainen H, Pennanen T O, Vaara J (2009). Can J Chem.

[65] Martin B, Autschbach J (2016). Phys Chem Chem Phys.

[66] Clérac R, Cotton F A, Daniels L M, Dunbar K R, Murillo C A, Pascual I (2000). Inorg Chem.

[67] Thomas B J, Mitchell J F, Theopold K H, Leafy J A (1988). J Organomet Chem.

[68] Emrich R, Heinemann O, Jolly P W, Krüger C, Verhovnik G P J (1997). Organometallics.

[69] Monillas W H, Yap G P A, MacAdams L A, Theopold K H (2007). J Am Chem Soc.

[70] Wink D J, Wang N F, Creagan B T (1989). Organometallics.

[71] Fischer H, Hofmann J (1991). Chem Ber.

[72] Noh S K, Sendlinger S C, Janiak C, Theopold K H (1989). J Am Chem Soc.

[73] Richeson D S, Mitchell J F, Theopold K H (1989). Organometallics.

[74] Messere R, Spirlet M-R, Jan D, Demonceau A, Noels A F (2000). Eur J Inorg Chem.

[75] Nicoara C (2006). Neue Cyclopentadienyl ~ N-Donor – Liganden für Chrom-basierte Single-Site-Katalysatoren zur Olefinpolymerisation.

[76] Kirillov E, Roisnel T, Razavi A, Carpentier J-F (2009). Organometallics.

[77] Smith G M, Palmaka S W, Rogers J S, Malpass D B (1998). Polyalkylaluminoxane compositions formed by non-hydrolytic means. U.S. Patent.

[78] Busico V, Cipullo R, Cutillo F, Friederichs N, Ronca S, Wang B (2003). J Am Chem Soc.

