# Supplementary material for: Molecular weight control in organochromium olefin polymerization catalysis by hemilabile ligand–metal interactions
Source: Beilstein J Org Chem. 2016 Jul 4;12:1372–9. doi: 10.3762/bjoc.12.131 (PMC4979879; doi:10.3762/bjoc.12.131)
Supplement: File 1 — Experimental part. [file Beilstein_J_Org_Chem-12-1372-s001.pdf]

Supporting Information

for

Molecular weight control in organochromium olefin polymerization catalysis by hemilabile ligand–metal interactions

Stefan Mark, Hubert Wadepohl and Markus Enders*

Anorganisch-Chemisches Institut, Heidelberg University, Im Neuenheimer Feld 270, D-69120 Heidelberg, Germany

Email: Markus Enders - markus.enders@uni-heidelberg.de

*Corresponding author

In memory of Professor Dr. Peter Hofmann.

Experimental part

Contents:

Experimental procedures

Table S1 with details of X-ray structure analysis

Molecular pictures of molecules analyzed by X-rax diffraction

Experimental procedures

Materials and general considerations

Unless noted otherwise, all manipulations were carried out under inert argon or nitrogen atmosphere using standard Schlenk techniques. All glassware was heated and dried under vacuum before use. Toluene, tetrahydrofuran (THF), dichloromethane and *n*-hexane were dried using a solvent purifier system based on molecular sieves supplied by VAC and were degassed prior to use. Ethylene with a purity grade of 99.95% was used as received. 2,3,4-Trimethyl-1-(8-quinolyl)cyclopentadiene and dichloro-η^5-[3,4,5-trimethyl-1-(8-quinolyl)-2-(trimethylsilyl)cyclopentadienyl]chromium(III) [1] as well as the chromium precursor $CrCl_3(THF)_3$ [2] were synthesized according to literature procedures. Potassium hydride was purchased as 30 wt % suspension in mineral oil from Sigma-Aldrich, washed thoroughly with dry *n*-hexane and stored as powder under inert gas. All other reagents were commercial grade.

NMR spectra were recorded on a Bruker DRX 200, Bruker Avance II 400 or Bruker Avance III 600 spectrometer. ^{1}H and ^{13}C NMR chemical shifts were referenced to the residual solvent signal. NMR assignments were confirmed by H,H-COSY, HSQC and HMBC experiments. Mass spectra were recorded on a Finnigan MAT8230 and a Jeol JMS-700 spectrometer. Elemental analyses were performed on a CHN-O-Rapid (Heraeus) by the Mikroanalytisches Labor, Organisch-Chemisches Institut, University of Heidelberg. Size exclusion chromatography (SEC) was carried out in 1,2,4-trichlorobenzene at 150 °C at a flow rate of 1 mL min^{-1} on a Polymer Laboratories 220 instrument equipped with Olexis columns. Data reported were determined vs polystyrene standards. Differential scanning calorimetry (DSC) was performed at a heating rate of 10 K min^{-1} on a Mettler Toledo DSC821^e using the software STARe. The reported DSC data are from the second heating cycles. Polymer crystallinity was calculated based on a melt enthalpy of 289 J g^{-1} for 100 % crystalline polyethylene [3].

X-ray crystal structure determinations

Crystal data and details of the structure determinations are compiled in Table S1. Full shells of intensity data were collected at low temperature (100(1) K) with a Bruker AXS Smart 1000 CCD diffractometer (Mo Kα radiation, sealed X-ray tube, graphite monochromator, λ = 0.71073 Å). Data were corrected for air and detector absorption, Lorentz and polarization effects [4]; absorption by the crystal was treated with a semiempirical multiscan method [5-7]. The structures were solved by conventional direct methods (compound **L3**) [8,9] or by the heavy atom method combined with structure expansion by direct methods applied to difference structure factors (all other structures) [10,11] and refined by full-matrix least squares methods based on F^2 against all unique reflections [8,12,13]. All non-hydrogen atoms were given anisotropic displacement parameters. Hydrogen atoms were generally input at calculated positions and refined with a riding model. When justified by the quality of the data the positions of some hydrogen atoms were taken from difference Fourier syntheses and refined. The disordered toluene solvent in **4**·0.5 toluene was subjected to suitable geometry constraints/restraints (rigid hexagon with the methyl group carbon atom restrained to be in the plane and to bisect the external CCC angle of the phenyl ring) and adp restraints (rigid body approximation). Crystals of **8** were multiples. After multicomponent integration data were extracted for the major component [14] and only these were used to solve and refine the structure.

CCDC 1452756–1452761 contains the supplementary crystallographic data for this paper. These data can be obtained free of charge from The Cambridge Crystallographic Data Centre via www.ccdc.cam.ac.uk/data_request/cif.

Computational details

Density functional theory calculations were carried out using the Gaussian 03 program package [15]. Geometries have been fully optimized using the B3LYP functional [16] on a 6-311g* basis set level [17]. The obtained energy values were characterized as minima by analyzing the frequency values with regard to the absence of zero or imaginary frequencies. All energy values shown are zero point vibrational corrected energies.

General numeration

The numeration of the atoms of the quinolyl ligand part for the assignment of both ^{1}H and ^{13}C NMR signals is shown below:

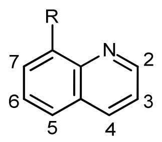

General procedure for the syntheses of the ligands

The syntheses of all new ligands was performed according to a modified literature procedure for the synthesis of 3,4,5-trimethyl-1-(8-quinolyl)-2-(trimethylsilyl)cyclopentadiene [1]. To a suspension of potassium hydride in THF 0.90–1.00 equivalents of 2,3,4-trimethyl-1-(8-quinolyl)cyclopentadiene were added slowly, accompanied by a change of color from yellow to dark violet. After stirring overnight 1.05–1.20 equivalents of an appropriate chlorodimethylsilane were added followed by continuous stirring overnight. The product mixture was quenched with iced water/NH_4Cl, washed with water, extracted with diethyl ether and the ethereal solution was dried over $MgSO_4$. The raw product was purified by column chromatography using dichloromethane as solvent and silica as stationary phase. The products are highly viscous orange oils.

3,4,5-Trimethyl-1-(8-quinolyl)-2-[dimethyl[3,5-bis(trifluoromethyl)phenyl]silyl]-cyclopentadiene (L2)

Quantities: 0.10 g ($2.5 \cdot 10^{-3}$ mol) potassium hydride, 0.60 g ($2.5 \cdot 10^{-3}$ mol) 2,3,4-trimethyl-1-(8-quinolyl)cyclopentadiene, 0.80 g ($2.6 \cdot 10^{-3}$ mol) chlorodimethyl[3,5-bis(trifluoro-methyl)phenyl]silane, 40 mL THF. Yield: 38%. ^{1}H NMR (399.89 MHz, $CDCl_3$, 22 °C): δ = 0.00 (bs, 6 H, Si*Me*$_2$); 1.88 (s, 3 H, HCp*Me*); 1.91 (d, $^4J_{H,H}$ = 1.5 Hz, 3 H, HCp*Me*); 2.09 (s, 3 H, HCp*Me*); 4.81 (bs, 1 H, *H*C_{Cp}); 7.22 (dd, $^3J_{H,H}$ = 8.5 Hz, $^3J_{H,H}$ = 4.2 Hz, 1 H, *H3*); 7.24 (dd, $^3J_{H,H}$ = 8.0 Hz, $^4J_{H,H}$ = 1.4 Hz, 1 H, *H5/H7*); 7.30 (s, 2 H, *m*-C_{aryl}*H*); 7.34 (dd, $^3J_{H,H}$ = 8.0 Hz, $^3J_{H,H}$ = 7.2 Hz, 1 H, *H6*); 7.40 (s, 1 H, *p*-C_{aryl}*H*); 7.42 (dd, $^3J_{H,H}$ = 7.2 Hz, $^4J_{H,H}$ = 1.4 Hz, 1 H, *H5/H7*); 7.86 (dd, $^3J_{H,H}$ = 8.5 Hz, $^4J_{H,H}$ = 1.8 Hz, 1 H, *H4*); 8.78 (dd, $^3J_{H,H}$ = 4.2 Hz, $^4J_{H,H}$ = 1.8 Hz, 1 H, *H2*). ^{13}C NMR (100.56 MHz, $CDCl_3$, 22 °C): δ = -6.46 (Si*Me*); -2.05 (Si*Me*); 11.26 (HCp*Me*); 12.82 (HCp*Me*); 15.05 (HCp*Me*); 53.60 (HC_{Cp}); 120.76 (*C3*); 121.80 (*p*-C_{aryl}H); 125.77 (*C6*); 126.44 (*C5/C7*); 128.24 (*Cq*); 129.02 (*Cq*); 129.33 (*Cq*); 130.14 (*C5/C7*); 132.40 (*m*-C_{aryl}H); 135.42 (*Cq*); 135.73 (*C4*); 137.08 (*Cq*); 137.34 (*Cq*); 140.15 (*Cq*); 141.09 (*Cq*); 149.31 (*C2*); (^{13}C signal of the CF_3-groups not visible). ^{29}Si NMR (79.44 MHz, $CDCl_3$, 22 °C): δ = -1.74. ^{19}F NMR (376.23 MHz, $CDCl_3$, 22 °C): δ = -62.40. GC MS (EI): *m/z* : 505 (M^+), 490 (M^+-Me).

3,4,5-Trimethyl-1-(8-quinolyl)-2-[dimethyl(pentafluorophenyl)silyl]cyclopentadiene (L3)

Quantities: 0.57 g ($14.2 \cdot 10^{-3}$ mol) potassium hydride, 3.17 g ($13.5 \cdot 10^{-3}$ mol) 2,3,4-trimethyl-1-(8-quinolyl)cyclopentadiene, 3.69 g ($14.2 \cdot 10^{-3}$ mol) chlorodimethyl(pentafluorophenyl)silane, 120 mL THF. Yield: 46%. ^{1}H NMR (399.89 MHz, $CDCl_3$, 22 °C): δ = -0.06 (s, 3 H, Si*Me*); 0.46 (s, 3 H, Si*Me*); 2.02 (s, 3 H, HCp*Me*); 2.09 (d, $^4J_{H,H}$ = 1.5 Hz, 3 H, HCp*Me*); 2.17 (s, 3 H, HCp*Me*); 5.04 (bs, 1 H, *H*C_{Cp}); 7.35 (dd, $^3J_{H,H}$ = 8.3 Hz, $^3J_{H,H}$ = 4.2 Hz, 1 H, *H3*); 7.49 (m, 2 H, *H5/H7*); 7.54 (m, 1 H, *H6*); 8.00 (dd, $^3J_{H,H}$ = 8.3 Hz, $^4J_{H,H}$ = 1.8 Hz, 1 H, *H4*); 8.88 (dd, $^3J_{H,H}$ = 4.2 Hz, $^4J_{H,H}$ = 1.8 Hz,

1 H, *H2*). ^{13}C NMR (100.56 MHz, $CDCl_3$, 22 °C): δ = -6.64 (Si*Me*); -0.58 (Si*Me*); 11.48 (HCp*Me*); 12.99 (HCp*Me*); 14.78 (HCp*Me*); 53.40 (HC_{Cp}); 120.83 (*C3*); 125.91 (*C6*); 126.02 (*C5/C7*); 127.98 (*Cq*); 130.39 (*C5/C7*); 135.15 (*Cq*); 135.34 (*C4*); 136.51 (*Cq*); 136.56 (*Cq*); 137.79 (*Cq*); 140.47 (*Cq*); 147.09 (*Cq*); 149.45 (*C2*); (^{13}C-signals of the C_6F_5-group not visible). ^{29}Si NMR (79.44 MHz, $CDCl_3$, 22 °C): δ = -2.18. ^{19}F NMR (376.23 MHz, $CDCl_3$, 22 °C): δ = -162.28 (m, 2 F, *m-F*); -153.78 (m, 1 F, *p-F*); -125.57 (dd, $^3J_{F,F}$ = 26.8 Hz, $^4J_{F,F}$ = 8.9 Hz, 2 F, *o-F*). Anal. Calcd for $C_{25}H_{22}F_5NSi$: C 65.34, H 4.83, N 3.05. Found: C 64.38, H 4.68, N 2.95.

3,4,5-Trimethyl-1-(8-quinolyl)-2-[dimethyl(3,3,3-trifluoropropyl)silyl]cyclopentadiene (L4)

Quantities: 0.24 g ($6.0 \cdot 10^{-3}$ mol) potassium hydride, 1.40 g ($6.0 \cdot 10^{-3}$ mol) 2,3,4-trimethyl-1-(8-quinolyl)cyclopentadiene, 1.19 g ($6.2 \cdot 10^{-3}$ mol) chlorodimethyl(3,3,3-trifluoropropyl)silane, 60 mL THF. Yield: 41%. ^{1}H NMR (399.89 MHz, C_6D_6, 22 °C): δ = -0.47 (s, 3 H, Si*Me*); -0.34 (s, 3 H, Si*Me*); 0.33 (m, 2 H, SiC*H2*); 1.60 (m, 2 H, CF_3C*H2*); 1.87 (s, 3 H, HCp*Me*); 1.93 (d, $^4J_{H,H}$ = 1.5 Hz, 3 H, HCp*Me*); 1.97 (s, 3 H, HCp*Me*); 4.65 (bs, 1 H, HC_{Cp}); 6.77 (dd, $^3J_{H,H}$ = 8.3 Hz, $^3J_{H,H}$ = 4.2 Hz, 1 H, *H3*); 7.24 (dd, $^3J_{H,H}$ = 8.0 Hz, $^3J_{H,H}$ = 7.2 Hz, 1 H, *H6*); 7.36 (dd, $^3J_{H,H}$ = 8.0 Hz, $^4J_{H,H}$ = 1.4 Hz, 1 H, *H5/H7*); 7.43 (dd, $^3J_{H,H}$ = 7.2 Hz, $^4J_{H,H}$ = 1.4 Hz, 1 H, *H5/H7*); 7.58 (dd, $^3J_{H,H}$ = 8.3 Hz, $^4J_{H,H}$ = 1.8 Hz, 1 H, *H4*); 8.70 (dd, $^3J_{H,H}$ = 4.2 Hz, $^4J_{H,H}$ = 1.8 Hz, 1 H, *H2*). ^{13}C NMR (100.56 MHz, C_6D_6, 22 °C): δ = -4.20 (Si*Me*); -3.32 (Si*Me*); 6.33 (Si*C*H_2); 11.47 (HCp*Me*); 13.33 (HCp*Me*); 15.10 (HCp*Me*); 28.90 (q, $^2J_{C,F}$ = 28.6 Hz, CF_3*C*H_2); 54.43 (HC_{Cp}); 121.03 (*C3*); 126.27 (*C6*); 126.74 (*C5/C7*); 129.21 (*Cq*); 129.70 (*Cq*); 130.75 (*C5/C7*); 136.18 (*C4*); 136.63 (*Cq*); 137.48 (*Cq*); 138.65 (*Cq*); 139.86 (*Cq*); 148.02 (*Cq*); 149.55 (*C2*); (^{13}C signal of the CF_3-group not visible). ^{29}Si NMR (79.44 MHz, C_6D_6, 22 °C): δ = 2.76. ^{19}F NMR (376.23

MHz, C_6D_6, 22 °C): δ = -68.68 (t, $^3J_{F,H}$ = 10.4 Hz). GC MS (EI): *m/z*: 389 (M^+), 374 (M^+-Me), 292 (M^+-$CH_2CH_2CF_3$).

3,4,5-Trimethyl-1-(8-quinolyl)-2-(dimethylvinylsilyl)cyclopentadiene (L5)

Quantities: 0.43 g ($10.7\cdot10^{-3}$ mol) potassium hydride, 2.30 g ($9.8\cdot10^{-3}$ mol) 2,3,4-trimethyl-1-(8-quinolyl)cyclopentadiene, 1.43 g ($11.9\cdot10^{-3}$ mol) chlorodimethylvinylsilane, 120 mL THF. Yield: 61%. ^{1}H NMR (399.89 MHz, $CDCl_3$, 22 °C): δ = -0.44 (s, 3 H, Si*Me*); -0.33 (s, 3 H, Si*Me*); 2.00 (s, 3 H, HCp*Me*); 2.08 (d, $^4J_{H,H}$ = 1.4 Hz, 3 H, HCp*Me*); 2.12 (s, 3 H, HCp*Me*); 4.62 (bs, 1 H, HC_{Cp}); 5.37 (m, 1 H, Si*CH*CH_2); 5.68 (m, 2 H, SiCH*CH_2*); 7.39 (dd, $^3J_{H,H}$ = 8.3 Hz, $^3J_{H,H}$ = 4.2 Hz, 1 H, *H3*); 7.55 (m, 2 H, *H5/H7*); 7.74 (m, 1 H, *H6*); 8.12 (dd, $^3J_{H,H}$ = 8.3 Hz, $^4J_{H,H}$ = 1.8 Hz, 1 H, *H4*); 8.94 (dd, $^3J_{H,H}$ = 4.2 Hz, $^4J_{H,H}$ = 1.8 Hz, 1 H, *H2*). ^{13}C NMR (100.56 MHz, $CDCl_3$, 22 °C): δ = -5.37 (Si*Me*); -4.28 (Si*Me*); 11.32 (HCp*Me*); 12.85 (HCp*Me*); 15.06 (HCp*Me*); 54.80 (HC_{Cp}); 120.49 (*C3*); 125.88 (*C5/C7*); 126.02 (*C6*); 128.61 (*Cq*); 130.50 (*C5/C7*); 130.69 (SiCH*CH_2*); 135.71 (*Cq*); 135.79 (*Cq*); 135.96 (*C4*); 137.83 (*Cq*); 137.98 (*Cq*); 138.13 (Si*C*HCH_2); 139.16 (*Cq*); 147.98 (*Cq*); 149.31 (*C2*). GC MS (EI) *m/z*: 318 (M^+), 304 (M^+-Me), 292 (M^+-$CHCH_2$).

3,4,5-Trimethyl-1-(8-quinolyl)-2-(allyldimethylsilyl)cyclopentadiene (L6)

Quantities: 0.22 g ($5.5\cdot10^{-3}$ mol) potassium hydride, 1.18 g ($5.0\cdot10^{-3}$ mol) 2,3,4-trimethyl-1-(8-quinolyl)cyclopentadiene, 0.82 g ($6.1\cdot10^{-3}$ mol) allylchlorodimethylsilane, 80 mL THF. Yield: 49%. ^{1}H NMR (399.89 MHz, $CDCl_3$, 22 °C): δ = -0.42 (s, 3 H, Si*Me*); -0.34 (s, 3 H, Si*Me*); 1.08 (m, 2 H, Si*CH_2*); 2.00 (s, 3 H, HCp*Me*); 2.06 (d, $^4J_{H,H}$ = 1.4 Hz, 3 H, HCp*Me*); 2.15 (s, 3 H, HCp*Me*); 4.63 (m, 3 H, HC_{Cp} und $SiCH_2CH$*CH_2*); 5.44 (m, 1 H, $SiCH_2C$*H*); 7.41 (dd, $^3J_{H,H}$ = 8.3 Hz, $^3J_{H,H}$ = 4.2 Hz, 1 H, *H3*); 7.58 (m, 2 H, *H5/H7*); 7.76 (m, 1 H, *H6*); 8.12 (dd, $^3J_{H,H}$ = 8.3 Hz, $^4J_{H,H}$ = 1.8 Hz, 1 H, *H4*); 8.96 (dd, $^3J_{H,H}$ = 4.2 Hz,

$^{4}J_{H,H}$ = 1.8 Hz, 1 H, *H2*). ^{13}C NMR (100.56 MHz, $CDCl_3$, 22 °C): δ = -4.63 (Si*Me*); -4.13 (Si*Me*); 11.44 (HCp*Me*); 12.95 (HCp*Me*); 15.13 (HCp*Me*); 21.79 (SiCH_2); 54.46 (HC_{Cp}); 112.72 (SiCH_2CHCH_2); 120.70 (*C3*); 126.03 (*C5/C6/C7*); 126.20 (*C5/C6/C7*); 128.72 (*Cq*); 130.62 (*C5/C6/C7*); 134.33 (*Cq*); 135.06 (SiCH_2CH); 135.66 (*Cq*); 136.02 (*Cq*); 136.30 (*C4*); 137.94 (*Cq*); 139.39 (*Cq*); 147.68 (*Cq*); 149.39 (*C2*). ^{29}Si NMR (79.44 MHz, $CDCl_3$, 22 °C): δ = 1.85. GC MS (EI) *m/z*: 333 (M^+), 318 (M^+-Me), 292 (M^+-CH_2CHCH_2).

3,4,5-Trimethyl-1-(8-quinolyl)-2-(benzyldimethylsilyl)cyclopentadiene (L7)

Quantities: 0.42 g ($10.5\cdot10^{-3}$ mol) potassium hydride, 2.22 g ($9.4\cdot10^{-3}$ mol) 2,3,4-trimethyl-1-(8-quinolyl)cyclopentadiene, 2.11 g ($11.4\cdot10^{-3}$ mol) benzylchlorodimethylsilane, 120 mL THF. Yield: 54%. ^{1}H NMR (399.89 MHz, $CDCl_3$, 22 °C): δ = -0.54 (s, 3 H, Si*Me*); -0.29 (s, 3 H, Si*Me*); 1.63 (dd, $^{2}J_{H,H}$ = 46.5 Hz, $^{4}J_{H,H}$ = 13.2 Hz, 2 H, SiCH_2); 2.04 (s, 3 H, HCp*Me*); 2.09 (d, $^{4}J_{H,H}$ = 1.4 Hz, 3 H, HCp*Me*); 2.17 (s, 3 H, HCp*Me*); 4.66 (bs, 1 H, *H*C_{Cp}); 6.75 (d, $^{3}J_{H,H}$ = 7.4 Hz, 2 H, *o*-C_{aryl}*H*); 7.04 (pt, $^{3}J_{H,H}$ = 7.4 Hz, 1 H, *p*-C_{aryl}*H*); 7.14 (pt, $^{3}J_{H,H}$ = 7.4 Hz, 2 H, *m*-C_{aryl}*H*); 7.40 (dd, $^{3}J_{H,H}$ = 8.3 Hz, $^{3}J_{H,H}$ = 4.2 Hz, 1 H, *H3*); 7.49 (dd, $^{3}J_{H,H}$ = 7.2 Hz, $^{4}J_{H,H}$ = 1.2 Hz, 1 H, *H7*); 7.58 (pt, $^{3}J_{H,H}$ = 8.2 Hz, $^{3}J_{H,H}$ = 7.2 Hz, 1 H, *H6*); 7.75 (dd, $^{3}J_{H,H}$ = 8.2 Hz, $^{4}J_{H,H}$ = 1.2 Hz, 1 H, *H5*); 8.17 (dd, $^{3}J_{H,H}$ = 8.3 Hz, $^{4}J_{H,H}$ = 1.8 Hz, 1 H, *H4*); 8.96 (dd, $^{3}J_{H,H}$ = 4.2 Hz, $^{4}J_{H,H}$ = 1.8 Hz, 1 H, *H2*). ^{13}C NMR (100.56 MHz, $CDCl_3$, 22 °C): δ = -4.15 (Si*Me*); -3.75 (Si*Me*); 11.41 (HCp*Me*); 12.89 (HCp*Me*); 15.11 (HCp*Me*); 23.18(SiCH_2); 54.68 (HC_{Cp}); 120.58 (*C3*); 123.61 (*p*-C_{aryl}H); 125.92 (*C6*); 126.22 (*C5*); 127.80 (*m*-C_{aryl}H); 128.27 (*o*-C_{aryl}H); 128.68 (*Cq*); 130.45 (*C7*); 135.92 (*Cq*); 136.08 (*C4*); 136.12 (*Cq*); 137.82 (*Cq*); 138.06 (*Cq*); 139.33 (*Cq*); 140.06 (*Cq*); 147.86 (*Cq*); 149.45 (*C2*). GC MS (EI) *m/z*: 383 (M^+), 368 (M^+-Me), 292 (M^+-C_7H_7).

3,4,5-Trimethyl-1-(8-quinolyl)-2-[dimethyl(3-cyanopropyl)silyl]cyclopentadiene (L8)

Quantities: 0.37 g ($9.2\cdot10^{-3}$ mol) potassium hydride, 2.00 g ($8.5\cdot10^{-3}$ mol) 2,3,4-trimethyl-1-(8-quinolyl)cyclopentadiene, 1.66 g ($10.3\cdot10^{-3}$ mol) chlorodimethyl-(3-cyano)propylsilane, 120 mL THF. Yield: 36%. ^{1}H NMR (399.89 MHz, $CDCl_3$, 22 °C): δ = -0.33 (bs, 6 H, Si*Me₂*); -0.03 (m, 1 H, C*H₂*); 0.24 (m, 1 H, C*H₂*); 1.15 (m, 1 H, C*H₂*); 1.36 (m, 1 H, C*H₂*); 1.87 (m, 2 H, C*H₂*); 1.99 (s, 3 H, HCp*Me*); 2.07 (d, $^{4}J_{H,H}$ = 1.4 Hz, 3 H, HCp*Me*); 2.14 (s, 3 H, HCp*Me*); 4.60 (bs, 1 H, HC_{Cp}); 7.43 (dd, $^{3}J_{H,H}$ = 8.3 Hz, $^{3}J_{H,H}$ = 4.2 Hz, 1 H, *H3*); 7.58 (m, 2 H, *H6/H7*); 7.78 (dd, $^{3}J_{H,H}$ = 8.2 Hz, $^{4}J_{H,H}$ = 1.2 Hz, 1 H, *H5*); 8.21 (dd, $^{3}J_{H,H}$ = 8.3 Hz, $^{4}J_{H,H}$ = 1.8 Hz, 1 H, *H4*); 8.97 (dd, $^{3}J_{H,H}$ = 4.2 Hz, $^{4}J_{H,H}$ = 1.8 Hz, 1 H, *H2*). ^{13}C NMR (100.56 MHz, $CDCl_3$, 22 °C): δ = -4.86 (Si*Me*); -3.41 (Si*Me*); 11.38 (HCp*Me*); 12.87 (HCp*Me*); 14.09 (CH_2) 15.01 (HCp*Me*); 20.35(CH_2); 20.43 (CH_2); 53.92 (HC_{Cp}); 119.65 (*Cq*) 120.78 (*C3*); 126.22 (*C5*); 128.74 (*Cq*); 130.68 (*C7*); 135.48 (*Cq*); 136.23 (*Cq*); 136.37 (*C4/C6*); 137.77 (*Cq*); 137.99 (*Cq*); 139.73 (*Cq*); 147.41 (*Cq*); 149.39 (*C2*). GC MS (EI) *m/z*: 360 (M^+), 345 (M^+-Me), 292 (M^+-C_4H_6N).

General procedure for the synthesis of the complexes

The syntheses of the new complexes was performed according to a modified literature procedure for the synthesis of dichloro-η^5-[3,4,5-trimethyl-1-(8-quinolyl)-2-(trimethylsilyl)cyclopentadienyl]chromium(III) [1].

To a suspension of potassium hydride in THF equimolar amounts of an appropriate ligand (**L2**–**L8**) were added slowly, accompanied by a change of color from yellow to dark violet. After stirring overnight an equimolar amount of $CrCl_3(THF)_3$ was added followed by continuous stirring overnight. The solvent was removed under vacuum and the raw product was washed several times under inert gas with *n*-hexane and extracted with dichloromethane. The dichloromethane solution was centrifuged in order to remove insoluble impurities. The products are green blue powders.

Dichloro-η^5-[3,4,5-trimethyl-1-(8-quinolyl)-2-[dimethyl[3,5-bis(trifluoromethyl)phenyl]silyl]cyclopentadienyl]chromium(III) (2)

Quantities: 17.0 mg ($4.2 \cdot 10^{-4}$ mol) potassium hydride, 215.0 mg ($4.2 \cdot 10^{-4}$ mol) 3,4,5-trimethyl-1-(8-quinolyl)-2-[dimethyl[3,5-bis(trifluoromethyl)phenyl] silyl]cyclopentadiene (L2), 159.0 mg ($4.2 \cdot 10^{-4}$ mol) $CrCl_3(THF)_3$, 35 mL THF. Yield: 41%. ^{1}H NMR (399.89 MHz, C_6D_6, 22 °C): δ = -76 ($\nu_{1/2}$ = 3000 Hz, 1 H, *H2*); -56 ($\nu_{1/2}$ = 700 Hz, 1 H, *H4*); -30 ($\nu_{1/2}$ = 1300 Hz, 3 H, Cp*Me*); -28.28 (s, 3 H, Cp*Me*); -16.1 ($\nu_{1/2}$ = 150 Hz, 1 H, *H5*); 15.3 ($\nu_{1/2}$ = 53 Hz, 1 H, *H6*); 23 ($\nu_{1/2}$ = 850 Hz, 3 H, Cp*Me*); 51 ($\nu_{1/2}$ = 500 Hz, 1 H, *H3*). ^{19}F NMR (376.23 MHz, C_6D_6, 22 °C): δ = -62.7 ($\nu_{1/2}$ = 15 Hz); -62.4 ($\nu_{1/2}$ = 30 Hz). MS (EI) *m/z*: 626 (M^+), 591 (M^+-Cl), 543 (M^{++}-Me, -CF_3), 413 (M^+-$Ph(CF_3)_2$), 356 (M^+-$SiMe_2Ph(CF_3)_2$).

Dichloro-η^5-[3,4,5-trimethyl-1-(8-quinolyl)-2-[dimethyl(pentafluorophenyl)silyl]-cyclopentadienyl]chromium(III) (3)

Quantities: 0.23 g ($5.7 \cdot 10^{-3}$ mol) potassium hydride, 2.63 g ($5.7 \cdot 10^{-3}$ mol) 3,4,5-trimethyl-1-(8-quinolyl)-2-[dimethyl(pentafluorophenyl)silyl]cyclopentadiene (**L3**), 2.14 g ($5.7 \cdot 10^{-3}$ mol) $CrCl_3(THF)_3$, 120 mL THF. Yield: 21%. ^{1}H NMR (399.89 MHz, $CDCl_3$, 22 °C): δ = -53 ($\nu_{1/2}$ = 800 Hz, 1 H, *H4*); -35 ($\nu_{1/2}$ = 1000 Hz, 3 H, Cp*Me*); -32 ($\nu_{1/2}$ = 1000 Hz, 3 H, Cp*Me*); -14 ($\nu_{1/2}$ = 300 Hz, 1 H, *H5*); 17 ($\nu_{1/2}$ = 250 Hz, 1 H, *H6*); 22 ($\nu_{1/2}$ = 800 Hz, 3 H, Cp*Me*); 52 ($\nu_{1/2}$ = 700 Hz, 1 H, *H3*). ^{19}F NMR (376.23 MHz, $CDCl_3$, 22 °C): δ = -160 ($\nu_{1/2}$ = 180 Hz); -149 ($\nu_{1/2}$ = 180 Hz); -126 ($\nu_{1/2}$ = 180 Hz). MS (EI) *m/z*: 580 (M^+), 545 (M^+-Cl), 356 (M^+-$SiMe_2C_6F_5$).

Dichloro-η^5-[3,4,5-trimethyl-1-(8-quinolyl)-2-[dimethyl(3,3,3-trifluoropropyl)silyl]-cyclopentadienyl]chromium(III) (4)

Quantities: 0.096 g ($2.4\cdot10^{-3}$ mol) potassium hydride, 0.93 g ($2.4\cdot10^{-3}$ mol) 3,4,5-trimethyl-1-(8-quinolyl)-2-[dimethyl(3,3,3-trifluoropropyl)silyl]cyclopentadiene (**L4**), 0.895 g ($2.4\cdot10^{-3}$ mol) $CrCl_3(THF)_3$, 60 mL THF. Yield: 76%. ^1H NMR (399.89 MHz, $CDCl_3$, 22 °C): δ = -75 ($\nu_{1/2}$ = 2600 Hz, 1 H, *H2*); -55 ($\nu_{1/2}$ = 1200 Hz, 1 H, *H4*); -35 ($\nu_{1/2}$ = 2200 Hz, 6 H, Cp*Me*); -16 ($\nu_{1/2}$ = 300 Hz, 1 H, *H5*); 16 ($\nu_{1/2}$ = 150 Hz, 1 H, *H6*); 21 ($\nu_{1/2}$ = 1100 Hz, 3 H, Cp*Me*); 51 ($\nu_{1/2}$ = 700 Hz, 1 H, *H3*). ^{19}F NMR (376.23 MHz, $CDCl_3$, 22 °C): δ = -68.1 ($\nu_{1/2}$ = 85 Hz). MS (EI) *m/z*: 510 (M^+), 475 (M^+-Cl), 413 (M^+-$CH_2CH_2CF_3$), 356 (M^+-$SiMe_2CH_2CH_2CF_3$).

Dichloro-η^5-[3,4,5-trimethyl-1-(8-quinolyl)-2-(dimethylvinylsilyl)-cyclopentadienyl]chromium(III) (5)

Quantities: 0.13 g ($3.1\cdot10^{-3}$ mol) potassium hydride, 1.00 g ($3.1\cdot10^{-3}$ mol) 3,4,5-trimethyl-1-(8-quinolyl)-2-(dimethylvinylsilyl)cyclopentadiene (**L5**), 1.17 g ($3.1\cdot10^{-3}$ mol) $CrCl_3(THF)_3$, 60 mL THF. Yield: 3 %. ^1H NMR (399.89 MHz, $CDCl_3$, 22 °C): δ = -77 ($\nu_{1/2}$ = 3000 Hz, 1 H, *H2*); -57 ($\nu_{1/2}$ = 1200 Hz, 1 H, *H4*); -35 ($\nu_{1/2}$ = 2000 Hz, 3 H, Cp*Me*); -29 ($\nu_{1/2}$ = 1600 Hz, 3 H, Cp*Me*); -17 ($\nu_{1/2}$ = 350 Hz, 4 H, *H5*); 15.7 ($\nu_{1/2}$ = 150 Hz, 1 H, *H6*); 21 ($\nu_{1/2}$ = 1000 Hz, 3 H, Cp*Me*); 51 ($\nu_{1/2}$ = 700 Hz, 1 H, *H3*). MS (EI) *m/z*: 440 (M^+), 425 (M^+-CH_3), 405 (M^+-Cl).

Dichloro-η^5-[3,4,5-trimethyl-1-(8-quinolyl)-2-(benzyldimethylsilyl)-cyclopentadienyl]chromium(III) (7)

Quantities: 0.11 g ($2.7\cdot10^{-3}$ mol) potassium hydride, 1.09 g ($2.8\cdot10^{-3}$ mol) 3,4,5-trimethyl-1-(8-quinolyl)-2-(benzyldimethylsilyl)cyclopentadiene (**L7**), 1.06 g ($2.8\cdot10^{-3}$ mol)

$CrCl_3(THF)_3$, 60 mL THF. Yield: 76 %. ^{1}H NMR (399.89 MHz, CD_2Cl_2, 22 °C): δ = -76 ($\nu_{1/2}$ = 3000 Hz, 1 H, *H2*); -57 ($\nu_{1/2}$ = 1200 Hz, 1 H, *H4*); -43 ($\nu_{1/2}$ = 1600 Hz, 3 H, Cp*Me*); -23 ($\nu_{1/2}$ = 1400 Hz, 3 H, Cp*Me*); -17 ($\nu_{1/2}$ = 250 Hz, 4 H, *H5*); 15.4 ($\nu_{1/2}$ = 80 Hz, 1 H, *H6*); 17 ($\nu_{1/2}$ = 1000 Hz, 3 H, Cp*Me*); 51 ($\nu_{1/2}$ = 700 Hz, 1 H, *H3*). MS (EI) *m/z*: 504 (M^+), 489 (M^+-CH_3), 469 (M^+-Cl), 413 (M^+-Benzyl).

Dichloro-η^5-[3,4,5-trimethyl-1-(8-quinolyl)-2-(allyldimethylsilyl)-cyclopentadienyl]chromium(III) (6)

Quantities: 0.093 g ($2.3\cdot10^{-3}$ mol) potassium hydride, 0.776 g ($2.3\cdot10^{-3}$ mol) 3,4,5-trimethyl-1-(8-quinolyl)-2-(allyldimethylsilyl)cyclopentadiene (**L6**), 0.871 g ($2.3\cdot10^{-3}$ mol) $CrCl_3(THF)_3$, 80 mL THF. Yield: 81%. ^{1}H NMR (199.92 MHz, C_6D_6, 22 °C): δ = -83 ($\nu_{1/2}$ = 2400 Hz, 1 H, *H2*); -59 ($\nu_{1/2}$ = 500 Hz, 1 H, *H4*); -34 ($\nu_{1/2}$ = 1000 Hz, 3 H, Cp*Me*); -18 ($\nu_{1/2}$ = 250 Hz, 4 H, *H5* und Cp*Me*); 14.6 ($\nu_{1/2}$ = 70 Hz, 1 H, *H6*); 20 ($\nu_{1/2}$ = 800 Hz, 3 H, Cp*Me*); 50 ($\nu_{1/2}$ = 300 Hz, 1 H, *H3*). MS (EI) *m/z*: 454 (M^+), 413 (M^+-CH_2CHCH_2), 356 (M^+-$SiMe_2CH_2CHCH_2$).

Dichloro-η^5-[3,4,5-trimethyl-1-(8-quinolyl)-2-[dimethyl(3-cyanopropyl)silyl]-cyclopentadienyl]chromium(III) (8)

Quantities: 0.099 g ($2.5\cdot10^{-3}$ mol) potassium hydride, 0.894 g ($2.5\cdot10^{-3}$ mol) 3,4,5-trimethyl-1-(8-quinolyl)-2-[dimethyl(3-cyanopropyl)silyl]cyclopentadiene (**L8**), 0.929 g ($2.5\cdot10^{-3}$ mol) $CrCl_3(THF)_3$, 60 mL THF. Yield: 28%. ^{1}H NMR (399.89 MHz, C_7D_8, 22 °C): δ = -77 ($\nu_{1/2}$ = 3000 Hz, 1 H, *H2*); -59 ($\nu_{1/2}$ = 1200 Hz, 1 H, *H4*); -36 ($\nu_{1/2}$ = 2000 Hz, 3 H, Cp*Me*); -18 ($\nu_{1/2}$ = 300 Hz, 4 H, *H5*); -15 ($\nu_{1/2}$ = 1500 Hz, 3 H, Cp*Me*); 15 ($\nu_{1/2}$ = 150 Hz, 1 H, *H6*); 24 ($\nu_{1/2}$ = 1600 Hz, 3 H, Cp*Me*); 51 ($\nu_{1/2}$ = 600 Hz, 1 H, *H3*). MS (EI) *m/z*: 481 (M^+), 446 (M^+-Cl), 413 (M^+-Propyl-CN), 356 (M^+-$SiMe_2$Propyl-CN).

Ethylene polymerizations

The co-catalyst PMAO was supplied by AKZO-NOBEL as 7 wt % solution in toluene and was used as received. All polymerizations were carried out at atmospheric pressure and room temperature.

A 250 mL Schlenk flask, equipped with an output flow meter and cooled with a water bath, was filled with toluene (140 mL). In a 25 mL flask 2–10 μmol of the appropriate catalyst precursor **1**–**8** were dissolved in 10 mL of toluene and activated with PMAO. The Cr:Al ratio was 1:1000. After 5 min the catalyst solution was transferred to the prepared toluene flask, immediately followed by ethylene feeding via the Schlenk valve. The polymerization was operated 10–20 min under intense stirring, while ethylene gas was fed through a flow meter into the flask. The surplus of unreacted ethylene gas was measured with a second flow meter. The reaction was terminated by the addition of methanol/HCl (50 mL). The precipitated polyethylene was filtered, stirred in acetone for two hours and dried at 80 °C overnight to constant weight. The polymer was analyzed by differential scanning calorimetry (DSC) and high temperature size exclusion chromatography (SEC).

Table S1: Details of the crystal structure determinations of compounds **L3** and **4**–**8**.

	**L3**	**4**·0.5 toluene	**5**	**6**	**7**	**8**
formula	$C_{25}H_{22}F_5NSi$	$C_{25.5}H_{29}Cl_2CrF_3NSi$	$C_{21}H_{24}Cl_2CrNSi$	$C_{22}H_{26}Cl_2CrNSi$	$C_{26}H_{28}Cl_2CrNSi$	$C_{23}H_{27}Cl_2CrN_2Si$
crystal system	monoclinic	triclinic	triclinic	monoclinic	triclinic	triclinic
space group	$P2_1/c$	$P\bar{1}$	$P\bar{1}$	$P2_1/c$	$P\bar{1}$	$P\bar{1}$
a /Å	18.509(2)	8.8232(16)	8.740(5)	7.424(3)	10.341(4)	8.879(3)
b /Å	8.1642(10)	9.2882(17)	10.049(6)	15.632(6)	10.810(5)	14.149(4)
c /Å	15.5842(19)	17.850(3)	13.571(8)	19.003(9)	11.000(5)	20.470(6)
α /°		96.990(3)	99.575(16)		95.625(10)	72.960(5)
β /°	113.762(2)	95.950(3)	100.94(2)	100.666(12)	99.127(10)	81.171(6)
γ /°		114.035(3)	113.911(9)		97.038(9)	73.698(7)
V /Å^3	2155.3(5)	1307.0(4)	1029.8(10)	2167.3(16)	1196.3(9)	2352.9(13)
Z	4	2	2	4	2	4
M_r	459.52	557.49	441.40	455.43	505.48	482.45
F_{000}	952	576	458	948	526	1004
d_c /Mg·m^{-3}	1.416	1.417	1.424	1.396	1.403	1.362
μ /mm^{-1}	0.165	0.724	0.878	0.837	0.766	0.776
max., min. transmission factors	0.7464, 0.6457	0.7464, 0.6438	0.7464, 0.6520	0.7464, 0.6782	0.7464, 0.6819	0.7454, 0.5599
θ range /°	2.4 to 32.1	1.2 to 32.0	2.3 to 32.2	2.2 to 32.5	2.0 to 32.2	2.1 to 26.6
index ranges h,k,l	±27, ±12, ±22	±12, ±13, ±26	-12 ··· +13, ±14, -13 ··· +19	±10, ±22, ±28	-15 ··· +14, -16 ··· +15, ±16	-10 ··· +11, -16 ··· +17, ±25
reflections measured	50566	32068	14160	55440	30186	21969
unique [R_{int}]	7207 [0.0410]	8539 [0.0416]	6527 [0.0404]	7517 [0.0496]	7875 [0.0258]	9340 [0.149]
observed [$I \geq 2\sigma(I)$]	5506	6891	4636	6001	7133	5994
data / restraints /parameters	7207 / 0 / 320	8539 / 47 / 327	6527 / 0 / 267	7517 / 0 / 282	7875 / 0 / 324	9340 / 0 / 533
GooF on F^2	1.079	1.117	1.024	1.027	1.040	1.050
R indices [$F>4\sigma(F)$] $R(F)$, $wR(F^2)$	0.0435, 0.1065	0.0526, 0.1539	0.0417, 0.0910	0.0359, 0.0811	0.0279, 0.0757	0.0847, 0.1832
R indices (all data) $R(F)$, $wR(F^2)$	0.0664, 0.1274	0.0688, 0.1650	0.0747, 0.1064	0.0531, 0.0887	0.0323, 0.0801	0.1407, 0.2052
largest residual peaks /e·Å^{-3}	0.573, -0.241	1.046, -0.659	0.736, -0.427	0.756, -0.385	0.568, -0.328	1.214, -0.587

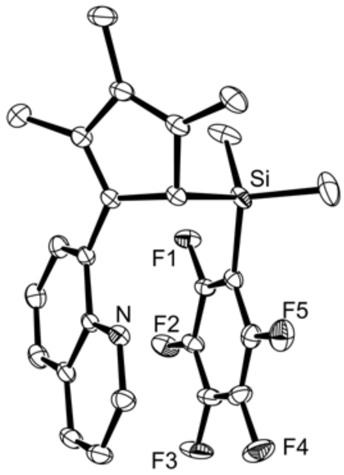

L3

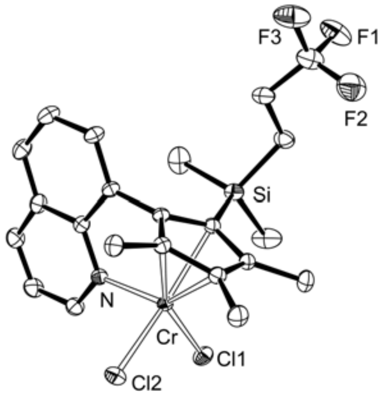

4

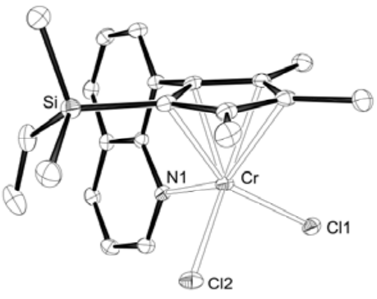

5

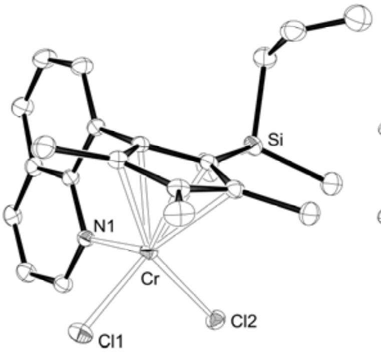

6

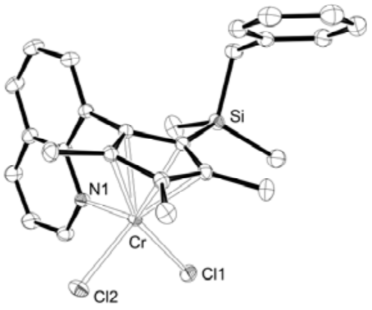

7

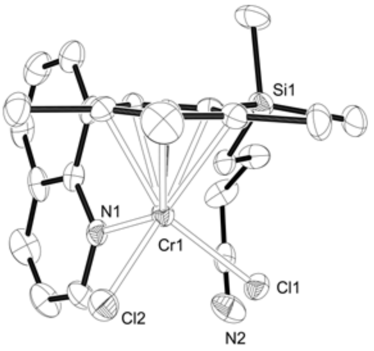

8

Figure S1: Molecular structures of the compounds analyzed by X-ray diffraction. Hydrogen atoms as well as co-crystallized solvent (**4**) or structure of a second independent molecule in the unit cell (**8**) are omitted for clarity.

References

[1] Fernández, P.; Pritzkow, H.; Carbó, J. J.; Hofmann, P.; Enders, M. *Organometallics* **2007**, *26*, 4402-4412.

[2] Herwig, W.; Zeiss, H. *J. Org. Chem.* **1958**, *23*, 1404-1404.

[3] Alamo, R. G.; Mandelkern, L. *Macromolecules* **1989**, *22*, 1273-1277.

[4] SAINT, Bruker AXS GmbH Karlsruhe, Germany, 1997-2013.

[5] Sheldrick, G. M.; SADABS Bruker AXS GmbH, Karlsruhe, Germany 2004-2014.

[6] Krause, L.; Herbst-Irmer, R.; Sheldrick, G. M.; Stalke, D. *J. Appl. Cryst.* **2015**, *48*, 3-10.

[7] Blessing, R. *Acta Cryst. A* **1995**, *51*, 33-38.

[8] Sheldrick, G. M.; SHELXS University of Göttingen, Germany, 1997.

[9] Sheldrick, G. *Acta Cryst. A* **2008**, *64*, 112-122.

[10] P. T. Beurskens In *Crystallographic Computing 3*; Sheldrick, G. M., Krüger, C., Goddard, R., Eds. Clarendon Press, Oxford, UK, 1985; p 216.

[11] Beurskens, P. T.; Beurskens, G.; Gelder, R. d.; Smits, J. M. M.; Garcia-Granda, S.; Gould, R. O.; DIRDIF-2008 Radboud University Nijmegen, The Netherlands, 2008.

[12] Sheldrick, G. *Acta Cryst. C* **2015**, *71*, 3-8.

[13] Sheldrick, G. M.; SHELXL-20xx; University of Göttingen and Bruker AXS GmbH: Karlsruhe, Germany, 2012-2014.

[14] Sheldrick, G. M.; TWINABS; Bruker AXS GmbH: Karlsruhe, Germany 2004-2012.

[15] Frisch, M. J.; Trucks, G. W.; Schlegel, H. B.; Scuseria, G. E.; Robb, M. A.; Cheeseman, J. R.; Montgomery, J., J. A.; Vreven, T.; Kudin, K. N.; Burant, J. C.; Millam, J. M.; Iyengar, S. S.; Tomasi, J.; Barone, V.; Mennucci, B.; Cossi, M.; Scalmani, G.; Rega, N.; Petersson, G. A.; Nakatsuji, H.; Hada, M.; Ehara, M.; Toyota, K.; Fukuda, R.; Hasegawa, J.; Ishida, M.; Nakajima, T.; Honda, Y.; Kitao, O.; Nakai, H.; Klene, M.; Li, X.; Knox, J. E.; Hratchian, H. P.; Cross, J. B.; Bakken, V.; Adamo, C.; Jaramillo, J.; Gomperts, R.; Stratmann, R. E.; Yazyev, O.; Austin, A. J.; Cammi, R.; Pomelli, C.; Ochterski, J. W.; Ayala, P. Y.; Morokuma, K.; Voth, G. A.; Salvador, P.; Dannenberg, J. J.; Zakrzewski, V. G.; Dapprich, S.; Daniels, A. D.; Strain, M. C.; Farkas, O.; Malick, D. K.; Rabuck, A. D.; Raghavachari, K.; Foresman, J. B.; Ortiz, J. V.; Cui, Q.; Baboul, A. G.; Clifford, S.; Cioslowski, J.; Stefanov, B. B.; Liu, G.; Liashenko, A.; Piskorz, P.; Komaromi, I.; Martin, R. L.; Fox, D. J.; Keith, T.; Al-Laham, M. A.; Peng, C. Y.; Nanayakkara, A.; Challacombe, M.; Gill, P. M. W.; Johnson, B.; Chen, W.; Wong, M. W.; Gonzalez, C.; Pople, J. A.; Gaussian, Inc.: Wallingford, CT, USA, 2004.

[16] Becke, A. D. *J. Chem. Phys.* **1993**, *98*.

[17] Lee, C.; Yang, W.; Parr, R. G. *Phys. Rev. B* **1988**, *37*, 785-789.
